# Feasibility and preliminary evidence of the immediate effect on balance, functionality, and cognition in adults over 50 through postural sway-meter training

**DOI:** 10.1371/journal.pone.0314357

**Published:** 2024-12-02

**Authors:** Plaiwan Suttanon, Wanadchapon Khanphed, Sairag Saadprai, Sudarat Apibantaweesakul

**Affiliations:** 1 Department of Physical Therapy, Faculty of Allied Health Sciences, Thammasat University, Pathum Thani, Thailand; 2 Thammasat University Research Unit in Health, Physical Performance, Movement, and Quality of Life for Longevity Society, Thammasat University, Pathum Thani, Thailand; 3 Department of Sports Science and Sports Development, Faculty of Allied Health Sciences, Thammasat University, Pathum Thani, Thailand; UFPE: Universidade Federal de Pernambuco, BRAZIL

## Abstract

An accelerometer-based device (postural sway-meter) is increasingly used for balance assessment, training, and fall prevention. However, limited information exists regarding its immediate effect on physical and cognitive performance, especially among middle-aged and older adults. This study aims to investigate the immediate effects of a balance training program using a postural sway-meter on balance, functional, and cognitive performance in adults over 50 years. This experimental study involved 72 participants aged 50 years and over, randomly assigned to either the intervention or control group. The intervention group underwent a 30-minute balance training session using the sway-meter with sound feedback (set at 75% of the stability limits at baseline). The control group participated in a 30-minute fall prevention knowledge session. The sway-meter measured balance during quiet stance and leaning. Functional performance was assessed using the multi-dimensional reach test (MDRT). Cognitive performance was evaluated through Trail Making Tests (TMT) A & B, as well as hand/foot reaction time assessments. Between-group comparisons at post-intervention showed no significant differences in balance, functional, and cognitive performance outcomes. Within-group analysis revealed a significant decrease in maximum reaching distance in the forward direction (MDRT) (p = 0.032, d = 0.31, 95% CI [-0.15, 0.78]) and the time to complete TMT-B (p = 0.036, d = 0.24, 95% CI [-0.22, 0.71]) in the intervention group. The control group showed a significant increase in COM angle sway excursion (lateral direction) (p = 0.011, d = 0.27, 95% CI [-0.19, 0.74]) and a decrease in TMT-A time (p = 0.031, d = 0.38, 95% CI [-0.09, 0.85]). Both groups significantly reduced hand reaction time (intervention: p = 0.036, d = 0.24, 95% CI [-0.22, 0.70]; control: p = 0.034, d = 0.20, 95% CI [-0.26, 0.66]) at post-intervention assessment. The findings of this study suggest that a single 30-minute balance training session using a postural sway-meter, delivered by a physiotherapist, is not only feasible and safe for community-dwelling older adults but also has the potential to significantly improve balance and cognitive outcomes. Enhancing the training program by increasing the amount of leaning and duration could further amplify these benefits, underscoring the need for a more robust training regimen.

## Introduction

Falling is a well-recognized health issue in older people, with one in three people aged over 65 living in the community falling each year [1, 2]. Recent research evidence supports that the fall rate has been increasingly reported since middle age [3].

Older adults exhibit reduced muscle strength and a slower postural stability response to falls, with a diminished ability to adapt and process sensory information during a fall [4, 5]. Importantly, age-related changes, including structural and functional deteriorations in musculoskeletal, nervous systems, and balance performance, have been observed since middle age [3, 6, 7]. This decline in postural stability has been noted to begin as early as 40–50 years of age [4].

Previous studies have used postural stability and leaning distance as outcomes for assessing fall risk and as an adjunct to fall warnings [5–7]. The Accelerometer based device is one of the assessment tools that have been developed to be used in assessing the balance ability as well as fall risk level [5–8]. Accelerometer was found to be of moderate to high accuracy in both healthy, unstable and those who have a walking impairment [6, 9, 10]. Previous studies showed that resistance and balance exercises could increase sedentary activity and physical fitness in older [11, 12].There are factors influencing the effectiveness of balance training programs including duration, intensity, types, and components of the program [13, 14]. The duration of balance exercise programs shown to effectively improve balance performance and/or reduce the risk of falls in older adults varies significantly, ranging from four weeks [15–17] to 6–12 months [18]. Additionally, the immediate or acute effects of a single session of an exercise program have also been documented [19, 20]. The acute effects of a single session of repeated training on balance disturbances while walking could improve stability during walking as well as improve cognitive performance in information processing speed and selective domains in healthy community-dwelling older adults [19, 20]. However, the evidence supporting these immediate effects remains limited. Several studies have demonstrated the positive effects of conventional exercise on walking and balance when combined with exercise technologies, such as wearable sensors in which an accelerometer is one of the most common devices used in exercise training purposes [21, 22]. A key advantage of wearable sensor devices is their ability to detect changes in balance and gait by measuring kinetic and kinematic movement data using inertial measurement units (IMUs) [22]. Furthermore, exercise training combined with wearable sensors and accelerometer-based devices offers additional benefits compared to conventional exercise alone. This includes feedback training, which can enhance motivation during exercise [22].

The postural sway meter is an example of an accelerometer-based device developed to detect COM sway angle during standing and leaning in which the high to very high correlation with the standardized 3D-Motion Analysis has been reported [10].

The literature review reveals a significant gap in knowledge regarding the immediate effects of balance training using an accelerometer-based device on both physical and cognitive performance, particularly in middle-aged and older adults. Therefore, this study aimed to evaluate the immediate effects of balance training with an accelerometer-based sway-meter on balance, functional, and cognitive performance in community-dwelling individuals aged 50 years and older.

## Materials and methods

### Study design

The study was an experimental study. The study protocol was approved by the Human Research Ethics Committees, Thammasat University (Project Number: 134/2564). Written informed consent was obtained from each participant. Healthy volunteers were prospectively recruited between May 2023 and March 2024.

### Participants

Adults aged 50 and over who had been living in the community were eligible for the study if they satisfied all of the following criteria: i) ability to stand from sitting, stand safely for at least 15 minutes, and walk outdoors with no more support than a single point stick; ii) ability to communicate and follow instructions iii) having no other serious or unstable health conditions (e.g. recent lower limb surgery, stroke with unilateral or bilateral paresis or Parkinson disease) that could restrict functional mobility. Those who had a cognitive impairment assessed by the Thai Mini-Mental State Examination [23] that could limit participation would be excluded.

**Fig 1:**
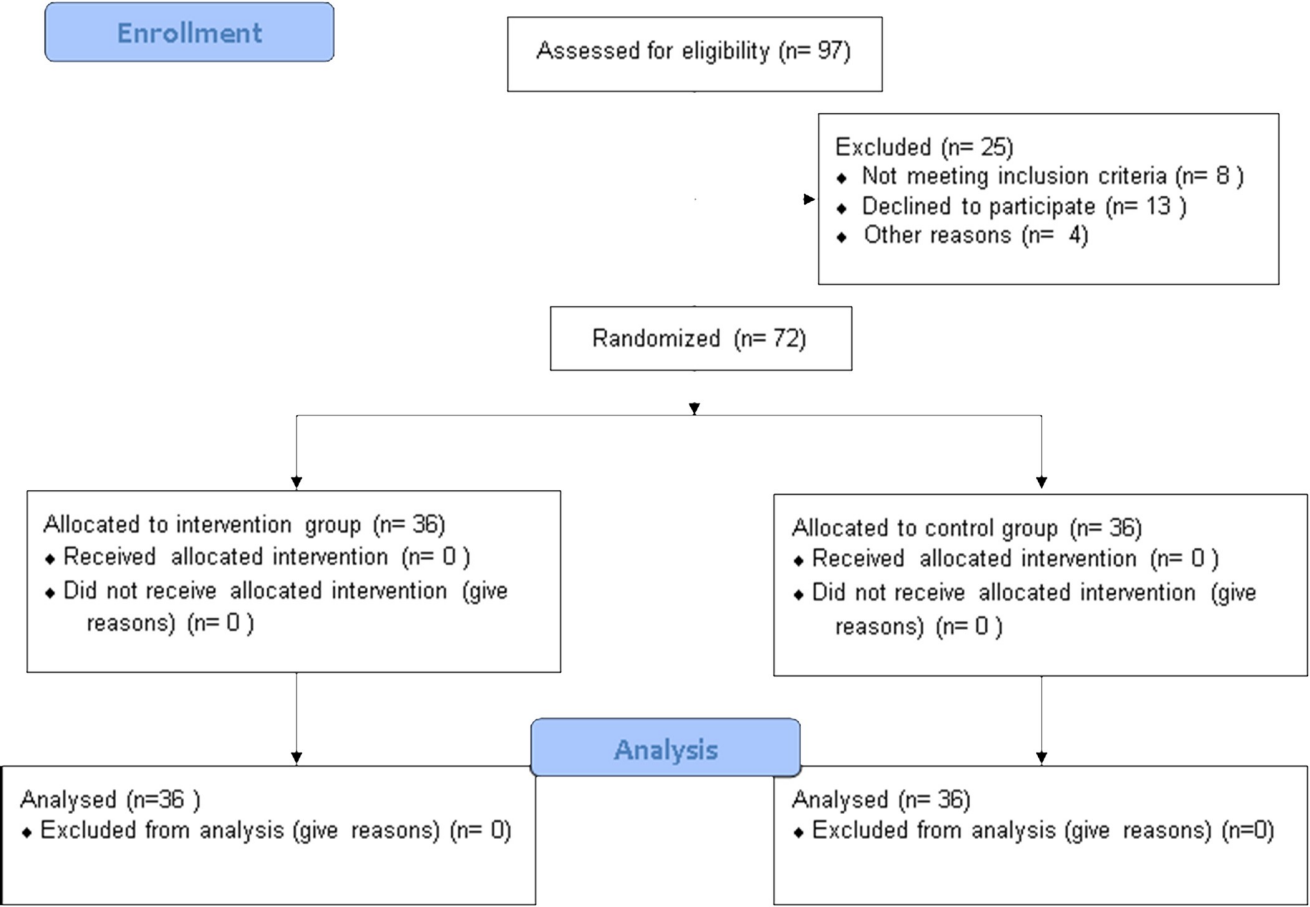
CONSORT diagram of the study.

Participants were recruited using a convenience sampling method. Each participant was randomly assigned to one of the two groups using a concealed randomization procedure: 1) the control group, which received a fall prevention knowledge session via video clip, or 2) the intervention group, which participated in a session of a balance training with the developed accelerometer-based sway-meter. Group allocation was determined using a computer-generated random number table and packed in opaque, sealed envelopes by a staff member who was not involved in the assessment processes. The CONSORT diagram is presented in Fig 1.

### Procedure

A baseline assessment was carried out, which included measures of cognitive performance, balance, and physical performance, respectively, and then repeated after a single session of the control or intervention programs (approximately 30 minutes) was completed. The **Activities-specific Balance Confidence (ABC) scale,** and the **International Physical Activity Questionnaire (IPAQ)** were used to assess participants’ characteristics at baseline. All measurements on both assessment occasions were undertaken by trained assessors who were physical therapists and blind to the group allocation.

### Intervention program

Participants assigned to the intervention group underwent a 30-minute session of balance training using the developed accelerometer-based sway-meter. The training protocol included the following components:

- **Warm-Up:** A 5-minute period involving lower extremity muscle stretching.
- **Balance Exercises:** A 20-minute session focusing on leaning in four different directions (anterior, posterior, left, and right) while standing in progressively challenging foot positions. These ranged from a stable stance with feet apart on a firm surface to a more balance-demanding tandem stance on a foam surface. Exercise intensity and progression were individualized based on each participant’s Limit of Stability (LOS), defined as the maximum degree of leaning excursion in the four directions measured during the baseline assessment. The progression criteria required participants to maintain a lean to 75%-80% of their LOS for three seconds without a loss of balance (e.g. taking a step) for six repetitions in each direction. Upon achieving this, the sway-meter was recalibrated to 90%-100% of the participant’s LOS, and training continued with six repetitions at this level, maintaining the 3-second hold in all four directions. These exercises were progressed from a stable stance with feet apart on a firm surface to a more challenging tandem stance on a foam surface. Progression varied for each participant, depending on individual performance.
- **Cool-Down:** A 5-minute period to conclude the session.

### Control program

Participants randomized to the control group received a 30-minute fall prevention knowledge session via a video clip.

### Measures

#### Balance and functional performance

- Balance assessment was conducted using a postural sway meter, with each condition assessed over three trials, and a two-minute break between each trial. The evaluation included five conditions: 1) quiet stance, 2) leaning forward, 3) leaning backward, and 4) leaning to the left, and leaning to the right. Postural sway was quantified by measuring center of mass (COM) angle displacement during each condition. Prior to testing, participants underwent a practice session. The average amount of postural sway during quiet stance measured from 3 trials was calculated. The average of maximum COM angle displacement during leaning 3 trials for each direction was used for data analyses [10].
- Multi-Directional Reach Test (MDRT) was employed as a functional assessment of individuals’ limits of stability in 4 directions: forward, backward, left, and right. Participants were instructed to reach as far as possible with their dominant arm raised to 90 degrees in each direction while standing with feet apart. The reaching distance (in centimeters) for each direction was recorded [24].

### Cognitive performance and reaction time

Cognitive performance was assessed using the Mini-Mental State Examination (MMSE) Thai version [25], and the Trail Making Test (TMT) [26].

Reaction time was measured using hand and foot reaction time tests. These tests recorded the time (in milliseconds) participants responded by touching a switch with their foot or hand after seeing a light signal. The dominant foot and hand were tested separately. The average reaction time was calculated from 20 trials for both foot and hand reaction time tests.

### Statistical analysis

Preliminary analysis evaluated the immediate effect of the intervention program (a session of balance training with the developed accelerometer-based sway-meter). Data were analysed using SPSS (IBM 23.0, SPSS Inc., Chicago, IL, USA). Descriptive statistics were used to describe the general characteristics of the participants. The Shapiro-Wilk test was employed to assess data distribution. For comparisons between the two groups at the post-intervention assessment time point, an Independent T-test was used for outcome measures with a normal distribution, while the Mann-Whitney U test was applied for outcome measures with a non-normal distribution. Within-group comparisons were performed using the Paired T-test (normally distributed data), and the Wilcoxon Signed Ranks test (non-normally distributed data). The level of statistical significance was assumed at p < 0.05. The Cohen’s d was used for effect size calculation for all the outcome measures of the study.

A power analysis was conducted for this study based on data from a previous study on the acute effects of balance training [19]. Using an estimated effect size of 0.5, the analysis indicated that 69 participants per group (a total of 138 participants) would be required to achieve 80% statistical power with a significance level (alpha) of 0.05.

## Results

### Participant characteristics

Seventy-two subjects were randomized into either the intervention group (n = 36) or the control group (n = 36). Baseline demographic and clinical characteristics, including global cognitive function, fear of falling, level of physical activity, balance, functional, and cognitive performance, are presented in Table 1.

**Table 1:** Characteristics of participants in intervention and control groups.

	Intervention Group (n = 36)	Control Group (n = 36)	p-value
**Age (Years)** ^**#**^	63.29 ± 6.68	62.50 ± 7.54	0.639
**Gender (Male/Female [n])** ^**##**^	9/27	7/29	0.586^a^
**Education (academic years)** ^**##**^			0.478
**≤ 6 years**	14	17	
**> 6 years**	22	19	
**Employment status (n)** ^**##**^			0.810^a^
** • Employment**	21	22	
** • Unemployment**	15	14	
**Medication conditions (n)** ^**##**^	1.56 ± 1.21	1.22 ± 1.20	0.238
**History of fall (n, past 12 months)** ^**##**^	4	6	0.496^a^
**Weight (kg.)** ^**#**^	59.74 ± 9.88	61.90 ± 12.23	0.492
**Height (cm.)** ^**##**^	156.89 ± 6.23	157.64 ± 9.19	0.946
**BMI (kg/m**^**2**^**)** ^**##**^	24.11 ± 3.30	25.14 ± 4.31	0.338
**Mini-Mental State Examination (MMSE) (score)** ^**##**^	25.78 ± 2.77	26.58 ± 2.09	0.306
**The Activities-specific Balance Confidence (ABC) scale (points)** ^**##**^	89.36 ± 10.11	87.92 ± 10.37	0.232
**International Physical Activity Questionnaire (IPAQ) (Level)** ^**##**^			0.424
** • Low**	9	5	
** • Moderate**	17	14	
** • High**	10	17	

Data reported are mean ± SD

a = Analysed by chi-square test

* = Significant difference (p<0.05).

^#^ Independent t-tests (normal distribution)

^##^ Mann-Whitney U test (non-normal distribution)

The average age of the participants was 62.89 ± 7.09 years, with 22.22% being male and 77.78% female. Approximately two-thirds of participants in both groups were still employed or engaged in occupational activities. Cognitive performance screening using the Mini-Mental State Examination (MMSE) showed scores ranging from 19 to 30 points. Participants had between 0 and 4 medical conditions, with hypertension being the most common. The Activities-Specific Balance Confidence (ABC) scale scores ranged from 61.25 to 100, with almost all participants (58 out of 72) classified as having a high level of function. There were no differences in the proportions of physical activity levels (low, moderate, and high) between the two groups, as measured by the International Physical Activity Questionnaire (IPAQ). There were no statistically significant differences in demographic and clinical characteristics between the two groups at baseline.

### Balance, functional, and cognitive performance

No statistically significant differences in balance, functional, and cognitive outcomes were found between the intervention and control groups at baseline

#### Comparisons of the outcome measures at post-intervention (between-group comparisons)

Comparisons of balance, functional, and cognitive performances between the two groups at the post-intervention assessment are presented in Table 2.

**Table 2:** Comparisons of mean difference in balance, functional and cognitive performance between the intervention and the control groups (at post-intervention assessment).

	Intervention Group (n = 36)	Control Group (n = 36)	p-value	Effect size (Cohen’s *d*)	95% CI
**Sway-Meter (COM angle (degree))**					
***During quiet standing***					
**Excursion**					
**Anteroposterior direction** ^**#**^	-0.04 ± 0.28	0.06 ± 0.15	0.499	0.45	[-0.91, 0.02]
**Lateral direction** ^**##**^	0.26 ± 0.78	0.02 ± 0.46	0.056	0.37	[-0.09, 0.84]
***During maximum leaning***					
**Forward Direction** ^**#**^	-0.43 ± 3.91	-1.20 ± 4.02	0.411	0.19	[-0.27, 0.66]
**Backward Direction** ^**#**^	0.07 ± 2.60	0.08 ± 1.75	0.983	0.05	[-0.47, 0.46]
**Left Direction** ^**#**^	-0.08 ± 1.94	-0.31 ± 1.22	0.541	0.14	[-0.32, 0.60]
**Right Direction** ^**#**^	0.24 ± 1.84	-0.16 ± 1.33	0.297	0.25	[-0.21, 0.71]
**Multidirectional Reach Test (MDRT) (cm.)**					
**Forward Direction** ^**#**^	-1.75 ± 4.72	0.01 ± 6.33	0.184	0.32	[-0.78, 0.15]
**Backward Direction** ^**#**^	0.28 ± 4.09	-0.71 ± 5.01	0.363	0.22	[-0.25, 0.68]
**Left Direction** ^**#**^	-0.94 ± 4.38	0.56 ± 3.75	0.125	0.37	[-0.83, 0.10]
**Right Direction** ^**#**^	0.55 ± 4.08	-0.25 ± 3.91	0.399	0.20	[-0.26, 0.66]
**Cognitive performance**					
**Trail Making test-A (seconds)** ^**#**^	-3.47 ± 22.15	-7.04 ± 17.85	0.455	0.18	[-0.29, 0.64]
**Trail Making test-B (seconds)** ^**##**^	-16.36 ± 61.32	-19.67 ± 87.85	0.323	0.04	[-0.42, 0.51]
**Trail Making test B-A (seconds)** ^**##**^	-12.66 ± 65.70	13.34 ± 81.01	0.223	0.35	[-0.82, 0.11]
**Reaction time**					
**Foot (milliseconds)** ^**#**^	-33.37 ± 123.54	-18.94 ± 124.75	0.623	0.12	[-0.58, 0.35]
**Hand (milliseconds)** ^**##**^	-23.62 ± 122.35	-22.68 ± 110.80	0.901	0.01	[-0.47, 0.45]

Data reported are mean difference ± SD; *Significant differences (p<0.05)

^#^Independent t-tests (normal distribution)

^##^ Mann-Whitney U test (non-normal distribution)

At the immediate post-intervention assessment, the comparisons between the two groups showed no statistically significant differences in balance or functional outcomes, as measured by the Sway-Meter or the Multidirectional Reach Test (MDRT). For example, in the anteroposterior direction during quiet standing, the intervention group had a mean difference of -0.04° (± 0.28), while the control group had a mean difference of 0.06° (± 0.15), with a p-value of 0.499 and a corresponding effect size, d = 0.45, 95% CI [-0.91, 0.02]. In the lateral direction during quiet standing, the p-value was 0.056, with an effect size, d = 0.37, 95% CI [-0.09, 0.84].

Regarding cognitive performance and reaction time outcomes, for the Trail Making Test (TMT) Part A, completion times ranged from 24.19 to 144.31 seconds in the intervention group and from 18.73 to 83.44 seconds in the control group, with a p-value of 0.455 and an effect size, d = 0.18, 95% CI [-0.29, 0.64]. For TMT Part B, completion times ranged from 51.65 to 437.6 seconds in the intervention group and from 59.38 to 398.24 seconds in the control group, with a p-value of 0.323 and an effect size, d = 0.04, 95% CI [-0.42, 0.51]. Regarding foot reaction time, the reaction times ranged from 335.00 to 850.00 milliseconds in the control group and from 209.35 to 901.4 milliseconds in the intervention group, with the p-value of 0.623, and the effect size, d = 0.12, 95% CI [-0.58, 0.35]. Hand reaction times ranged from 387.40 to 1029.11 milliseconds in the control group and from 481.22 to 893.12 milliseconds in the intervention group. The p-value was 0.901, with an effect size, d = 0.01, 95% CI [-0.47, 0.45]. Although no statistically significant differences were observed between groups, the effect sizes provide additional context for understanding the magnitude of the differences, which in most cases were small to moderate.

#### Comparisons of the outcome measures assessed at two-time points: Baseline and post-intervention assessments in each group (with-in-group comparisons)

*Intervention group*. The results of balance, functional, and cognitive performance measured at the baseline and after a training session of the intervention group are reported in Table 3.

**Table 3:** Comparisons of mean ± SD in balance, functional, and cognitive performance between baseline and post-intervention assessments of the intervention group.

	Intervention Group (n = 36)				
	Baseline	Immediate	p-value	Effect size (Cohen’s *d*)	95% CI
**Sway-Meter (COM angle (degree))**					
**During quiet standing**					
Excursion					
Anteroposterior direction^#^	1.55 ± 0.27	1.51 ± 0.27	0.384	0.15	[-0.31, 0.61]
Lateral direction^##^	3.01 ± 0.55	3.27 ± 0.88	0.050	0.35	[-0.82, 0.11]
**During maximum leaning**					
Forward Direction^##^	16.91 ± 5.14	16.48 ± 4.75	0.741	0.09	[-0.38, 0.55]
Backward Direction^##^	6.12 ± 3.04	6.19 ± 3.15	0.677	0.02	[-0.48, 0.44]
Left Direction^##^	6.11 ± 1.93	6.03 ± 1.63	0.747	0.04	[-0.42, 0.51]
Right Direction^##^	5.57 ± 1.88	5.81 ± 1.95	0.303	0.13	[-0.59, 0.34]
**Multidirectional Reach Test (MDRT) (cm.)**					
Forward Direction^#^	29.01 ± 5.59	27.26 ± 5.53	0.032*	0.31	[-0.15, 0.78]
Backward Direction^##^	14.28 ± 4.93	14.56 ± 4.39	0.544	0.06	[-0.52, 0.40]
Left Direction^#^	20.33 ± 4.14	19.39 ± 3.86	0.207	0.23	[-0.23, 0.70]
Right Direction^##^	18.81 ± 3.76	19.35 ± 3.56	0.818	0.15	[-0.61, 0.31]
**Trail making test**					
Trail Making A (seconds)^##^	48.72 ± 19.05	45.24 ± 22.35	0.252	0.17	[-0.30, 0.63]
Trail Making B (seconds)^##^	136.40 ± 65.95	120.03 ± 69.15	0.036*	0.24	[-0.22, 0.71]
Trail Making test B-A (seconds)^##^	85.17 ± 60.06	72.87 ± 64.02	0.118	0.20	[-0.26, 0.66]
**Reaction time**					
Foot (millisecond)^##^	571.70 ± 149.07	538.34 ± 131.29	0.148	0.24	[-0.23, 0.70]
Hand (millisecond)^##^	626.24 ± 113.80	602.62 ± 80.47	0.034*	0.24	[-0.22, 0.70]

Data reported are mean ± SD

*Significant differences (p<0.05)

^#^ Independent t-tests (normal distribution)

^##^ Mann-Whitney U test (non-normal distribution)

**Fig 2:**
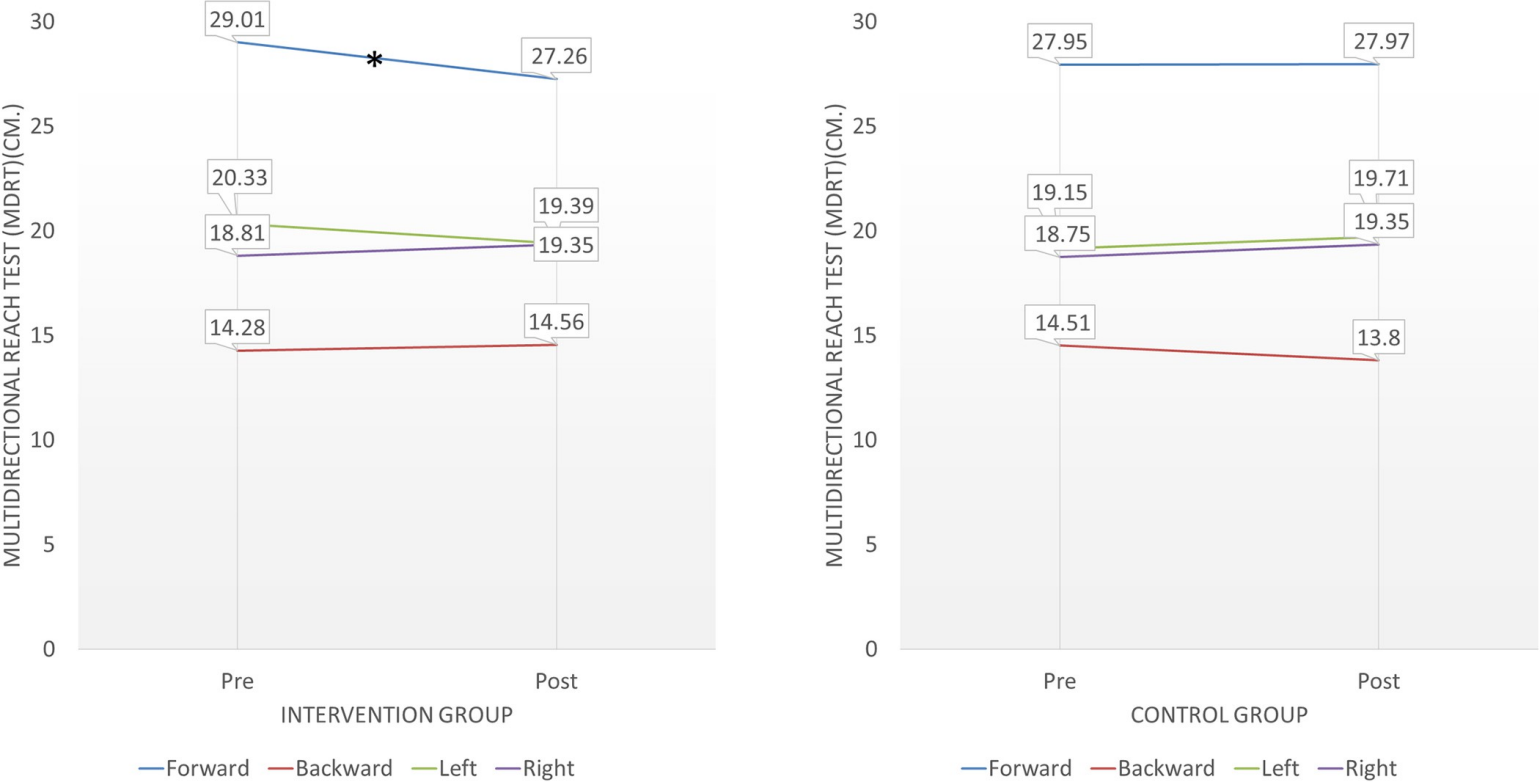
Comparisons of Multidirectional Reach Test (MDRT) between baseline and post-intervention assessments.

For the intervention group, the maximum reaching distance in the forward direction measured by the Multidirectional Reach Test (MDRT) significantly decreased after an exercise session with a sway meter (p = 0.032, d = 0.31, 95% CI [-0.15, 0.78]) (Table 3, Fig 2). This indicates a small-to-moderate effect size.

**Fig 3:**
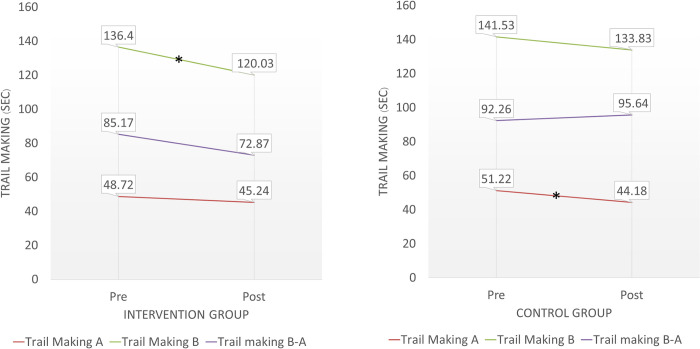
Comparisons of cognitive performance (Trail Making Test) between baseline and post-intervention assessments.

**Fig 4:**
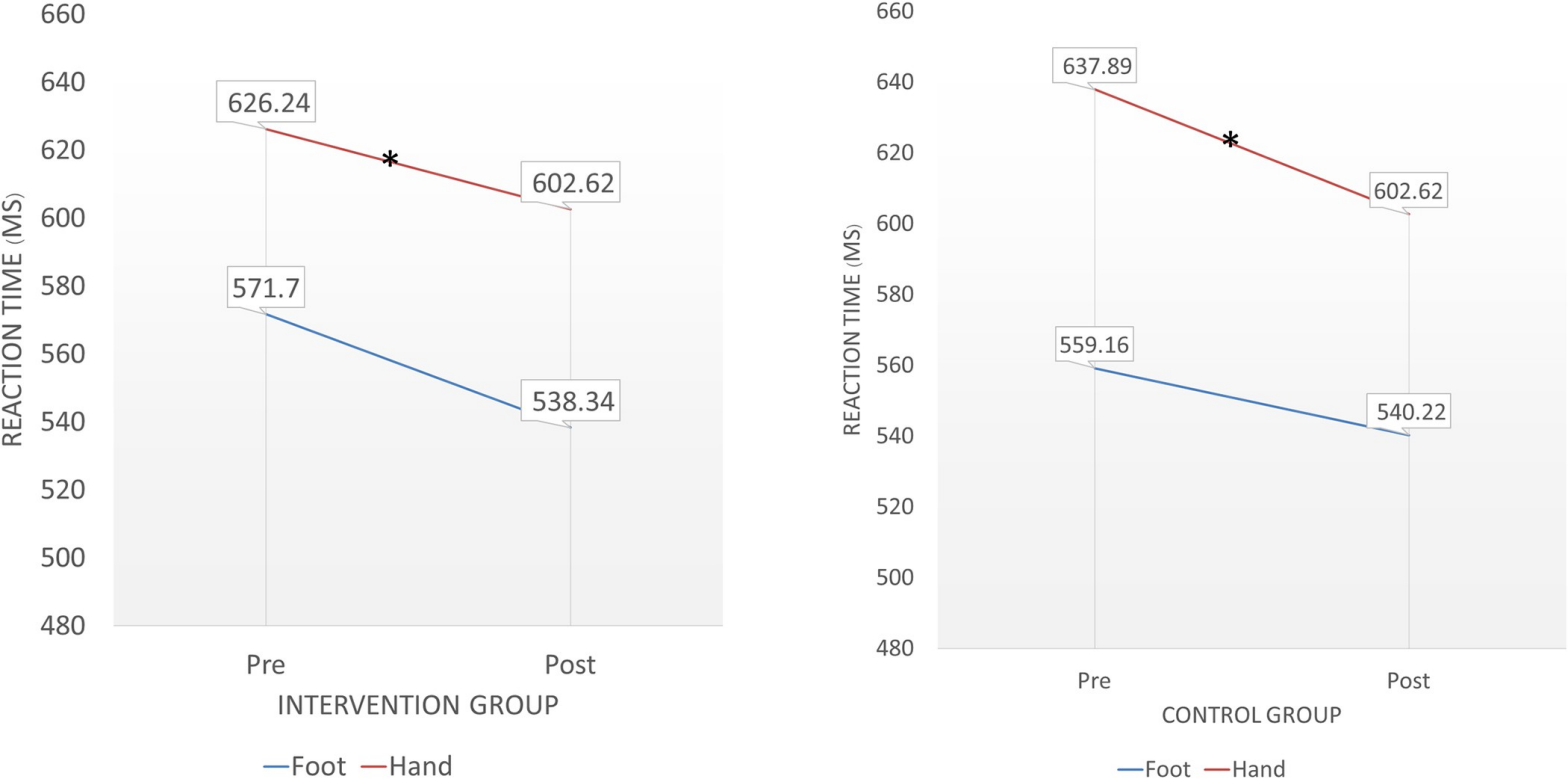
Comparisons of reaction time between baseline and post-intervention assessments.

Regarding the cognitive and reaction time outcome, a significant decrease in Trail Making Test B (p = 0.036, d = 0.24, 95% CI [-0.22, 0.71]) at the post-intervention assessment compared with baseline. Similarly, hand reaction time showed a significant improvement (p = 0.034, d = 0.24, 95% CI [-0.22, 0.70]) at post intervention (Table 3, Figs 3 and 4). Both outcomes suggest small effect sizes for these improvements.

For the control group, the COM angle sway excursion (in the left to right direction), which implies greater sway during quiet standing, was significantly increased at the post-intervention assessment (p = 0.011, d = 0.27, 95% CI [-0.19, 0.74]) (Table 4). Additionally, the time to complete Trail Making Test A was significantly decreased post-intervention compared with baseline (p = 0.031, d = 0.38, 95% CI [-0.09, 0.85]). Hand reaction time was also significantly decreased post-intervention (p = 0.037, d = 0.20, 95% CI [-0.26, 0.66]) (Table 4, Figs 3 and 4).

**Table 4:** Comparisons of mean ± SD in balance, functional, and cognitive performance between baseline and post-intervention assessments of the control group.

	Control Group (n = 36)				
	Baseline	Immediate	p-value	Effect size (Cohen’s *d*)	95% CI
**Sway-Meter (COM angle (degree))**					
**During quiet standing**					
Excursion					
Anteroposterior Distance^##^	1.18 ± 1.69	1.47 ± 0.45	0.789	0.23	[-0.70, 0.23]
Lateral Distance^##^	2.58 ± 1.95	3.18 ± 0.79	0.011*	0.27	[-0.19, 0.74]
**During maximum leaning**					
Forward Direction^#^	16.10 ± 5.43	14.90 ± 4.55	0.082	0.24	[-0.22, 0.70]
Backward Direction^##^	5.76 ± 2.33	5.84 ± 2.42	0.671	0.03	[-0.50, 0.43]
Left Direction^#^	5.99 ± 1.53	5.67 ± 1.59	0.132	0.21	[-0.26, 0.67]
Right Direction^#^	5.51 ± 1.93	5.35 ± 1.78	0.371	0.09	[-0.38, 0.55]
**Multidirectional Reach Test (MDRT) (cm.)**					
Forward Direction^#^	27.95 ± 4.87	27.97 ± 4.99	0.990	0.01	[-0.47, 0.46]
Backward Direction^#^	14.51 ± 4.52	13.80 ± 4.59	0.402	0.16	[-0.31, 0.62]
Left Direction^##^	19.15 ± 3.14	19.71 ± 3.57	0.405	0.17	[-0.63, 0.30]
Right Direction^##^	18.75 ± 4.11	18.50 ± 3.07	0.719	0.07	[-0.39, 0.53]
**Trail making test**					
Trail Making A (seconds)^##^	51.22 ± 21.27	44.18 ± 15.28	0.031*	0.38	[-0.09, 0.85]
Trail Making B (seconds)^##^	141.53 ± 117.09	133.83 ± 90.06	0.993	0.07	[-0.39, 0.54]
Trail Making test B-A (seconds)^##^	92.26 ± 79.93	95.64 ± 98.66	0.925	0.04	[-0.50, 0.42]
**Reaction time**					
Foot (millisecond)^#^	559.16 ± 128.38	540.22 ± 133.13	0.083	0.14	[-0.32, 0.61]
Hand (millisecond)^##^	637.89 ± 111.53	615.20 ± 115.91	0.037*	0.20	[-0.26, 0.66]

Data reported are mean ± SD

*Significant differences (p<0.05)

^#^ Independent t-tests (normal distribution)

^##^ Mann-Whitney U test (non-normal distribution)

### Safety and compliance with the intervention program

#### Balance training using the developed accelerometer-based sway-meter

There were no falls or injuries associated with performing the balance training using the developed accelerometer-based sway-meter. Only a few participants reported bodily discomfort, including dizziness (n = 1) and back pain (n = 2). However, those symptoms eased by the end of the day.

Full compliance (100%) was defined as a participant completing the 30-minute session of balance training using the developed accelerometer-based sway-meter. All of the 36 participants could complete the program.

#### Education and falls prevention booklet

The knowledge session was given in the form of video clips using a laptop and headphones. However, some participants (n = 4) preferred not to use headphones because they were not familiar with them. All 36 participants could complete the program session.

## Discussion

This study contributes valuable evidence that a session of balance training using a sway-meter, delivered by a physiotherapist, can be safely implemented in community-dwelling individuals aged 50 and over. Although the program did not yield significant improvements in balance, functional, and cognitive performance, the observed trends towards improvement in some outcome measures are encouraging. The absence of significant effects from a single 30-minute session suggests that a more prolonged intervention, involving multiple sessions of balance training with the sway-meter, might be necessary to achieve measurable benefits.

### Comparisons of outcome measures between the intervention and the control groups

At the post-intervention assessment, no statistically significant differences were found between the intervention and control groups, as measured by the sway-meter or the Multi-Directional Reach Test (MDRT). These findings suggest that a single session of balance training using the sway-meter may not be sufficient to significantly improve balance and functional performance. Additionally, most participants in this study were classified as young older adults (60–75 years) [27] without impaired balance, as evidenced by their MDRT scores [28].

The absence of significant differences between the groups in our study aligns with findings from Martelli et al. (2022) [19], which reported no differences between intervention and control groups. However, they observed improvements in balance performance within the intervention group compared to the baseline. Similarly, our study found changes between baseline and post-intervention within the intervention group. The lack of significant differences between the groups may be attributed to the small sample size, particularly when the outcome measures have high variability.

A systematic review and meta-analysis on the effect of real-time postural feedback in balance and mobility training in older adults [29] highlighted the benefits of sensory augmentation, such as vibrotactile and visual posture feedback, during balance training. Real-time feedback can assist participants make precise postural adjustments. In our study, however, feedback was limited to auditory cues indicating the body’s movement towards a limit point, which might not have been as effective in facilitating immediate improvements in balance training.

Regarding cognitive performance, no statistically significant differences were found between the groups. Dunsky’s study [30] demonstrated that a single session of aerobic exercise combined with balance and coordination exercises resulted in higher attention test scores compared to a control condition, measured by computerized cognitive tests. Compared to our study, the balance exercises in Dunsky’s study involved more challenging tasks, such as standing on one leg while focusing on a bouncing ball, which may have contributed to enhanced attention levels within 30 minutes. Additionally, Dunsky’s study utilized computerized cognitive tests specifically designed to assess attention. In contrast, our study measured cognitive performance using the Trail Making Test (TMT), in which attention is one of several domains of cognitive performance being assessed [31, 32].

Reaction time, another indicator of cognitive function, and information processing speed [33] did not show significant differences between groups in our study. This finding is consistent with Ferreira’s 2022 research [34], which found no significant improvements in verbal fluency, cognitive flexibility, or reaction times following a single session of augmented reality exergames or cycle ergometer exercise [34]. However, Roach’s 2014 study demonstrated that acute, intense exercise significantly improved reaction time, blood pressure, and heart rate [35]. The focus on aerobic exercise in Roach’s study, which increased blood flow and oxygen delivery to the brain and skeletal muscles, differs from our study, which focused on balance exercises.

### Comparisons of the outcome measures assessed at two-time points: Baseline and post-intervention assessments in each group (with-in group comparisons)

Our study demonstrates that the maximum reaching distance in the forward direction, as measured by the multidirectional reach test (MDRT), significantly decreased in the intervention group after a single session of balance training with the sway-meter (p = 0.032, d = 0.31, 95% CI [-0.15, 0.78]). This small-to-moderate effect size suggests that the observed decrease may have practical significance, reflecting neuroadaptive changes in the participants). This finding contrasts with previous research on the acute effects of perturbation-based balance training, which reported improvements in balance performance post-intervention [19]. One possible explanation for this discrepancy is the nature of the balance training programs: the previous study involved balance training under conditions of random external perturbation during walking, presenting a greater challenge compared to the static balance training in our study. Additionally, our sway-meter balance training focused on leaning in four directions (anterior-posterior and medial-lateral), primarily facilitating the use of the ankle movement strategy during standing and leaning.

The decreased maximal forward-reaching distance in the intervention group may be attributed to increased reliance on the ankle movement strategy following neuroadaptive changes, such as enhanced skill acquisition and increased muscle activation through motor unit synchronization and neural recruitment [36]. The MDRT, however, does not account for the specific movement strategies employed to maintain balance [28], potentially explaining the observed reduction in forward-reaching distance. It is plausible that the intervention group relied less on the hip strategy after training. Unfortunately, our study did not quantitatively record movement strategies during the MDRT, which is a limitation that should be addressed in future research.

In the control group, a significant increase in the center of mass (COM) angle sway excursion in the lateral direction was observed during standing (p = 0.011, d = 0.27, 95% CI [-0.19, 0.74]), indicating a small effect size. This increase in sway, though statistically significant, reflects a relatively small magnitude of change, which might suggest a measurement artifact or other external influences. This unexpected finding may be due to participants losing concentration during the quiet standing balance test, as it was the last assessment of the day. A similar trend was observed in the intervention group, suggesting that fatigue or attention lapses may have influenced the results.

Our study also revealed significant decreases in the time taken to perform the Trail Making Test (TMT) Part B in the intervention group (p = 0.036, d = 0.24, 95% CI [-0.22, 0.71]) and TMT Part A in the control group (p = 0.031, d = 0.38, 95% CI [-0.09, 0.85]) post-intervention. These small-to-moderate effect sizes suggest that the improvements, while statistically significant, were also of practical relevance. The TMT assesses attention, visual screening ability, and processing speed, serving as a good predictor of overall cognitive performance [32, 37, 38]. The improvement in TMT performance suggests that both intervention and control programs positively influenced cognitive function. The reduced time to complete TMT Part A in the control group may be attributed to the fall prevention knowledge session, which likely enhanced attention and processing speed through focused learning.

For the intervention group, the significant improvement in TMT Part B performance could be linked to the challenging balance exercises involving leaning in four directions and varying the base of support and surface stability. TMT Part B requires higher cognitive functions such as executive functioning, attention, planning, and organizing [32]. The demands of balance training likely stimulated these cognitive processes, leading to improved executive function.

Our exercise program incorporated both feedback and feedforward balance control mechanisms, engaging the brain to maintain balance and adapt to the training regimen. Reactive balance control relies on sensory feedback, while proactive balance control utilizes feedforward mechanisms to anticipate postural adjustments [39]. Participants likely used feedback control at the start of the exercise and transitioned to feedforward control during the training movements, enhancing their cognitive and balance capabilities.

The findings from the reaction time tests showed significant improvements in hand reaction time for both groups. In the control group, hand reaction time improved significantly (p = 0.037, d = 0.20, 95% CI [-0.26, 0.66]), indicating a small effect size. Similarly, the intervention group also showed improvements in hand reaction time, but these were likely influenced by a measurement-induced learning effect. This suggests that repeated testing can influence reaction time outcomes, highlighting the need to consider baseline learning effects in future assessments.

### Limitations and further study

One limitation of this study is that the balance training with the sway-meter incorporated self-perturbation exercises, with the maximum challenge set at 75% of each participant’s limit of stability measured at baseline. This intensity level may not have been sufficiently challenging to elicit significant improvements in balance performance, especially in the population of community-dwelling adults aged 50 years and older who had no balance impairments and remained active. Additionally, the small sample size is another limitation. While some trends of improvement were observed, a larger sample size could provide more statistical power to detect significant differences, particularly given the high variability in some of the outcome measures. Further research with a larger sample size is required to confirm and expand on our findings.

## Conclusion

A single 30-minute balance training session using a postural sway-meter, delivered by a physiotherapist, is feasible and safe for community-dwelling adults aged 50 and over. However, to enhance the program’s effectiveness in improving balance and cognitive performance, future interventions should consider increasing the intensity, incorporating real-time visual feedback, and providing greater challenges in balance exercises.
